# Delayed decrease in voriconazole concentration after resolution of inflammation in a patient with invasive pulmonary aspergillosis: a case report

**DOI:** 10.1186/s40780-026-00583-1

**Published:** 2026-05-18

**Authors:** Haruka Igarashi, Hayato Yokota, Ayano Saito, Yumiko Akamine, Naoto Takahashi, Masafumi Kikuchi

**Affiliations:** 1 Department of Pharmacy, Akita University Hospital, 1-1-1 Hondo, Akita, 010-8543 Japan; 2 Department of Hematology, Nephrology and Rheumatology, Akita University Graduate School of Medicine, Akita, Japan

**Keywords:** C-reactive protein, CYP2C19, Pulmonary aspergillosis, Trough plasma concentration, Voriconazole

## Abstract

**Background:**

Voriconazole (VRCZ) is an effective treatment for pulmonary aspergillosis. Active infectious disease leads to a reduction in cytochrome P450 activity, resulting in increased VRCZ concentrations. However, the impact of VRCZ dose reduction during the severe inflammatory state on VRCZ concentrations after the resolution of inflammation is not yet fully understood. Here, we report the time-course changes in VRCZ blood concentrations after inflammation in a patient with invasive pulmonary aspergillosis.

**Case presentation:**

A man in his 60s receiving oral VRCZ tablets at 400 mg/day for invasive pulmonary aspergillosis developed marked inflammation due to bacterial pneumonia and influenza virus infection. C-reactive protein (CRP) levels increased to a peak of 35.54 mg/dL, with a concomitant elevation in the VRCZ trough concentration to 4.7 µg/mL. Following dose reduction to VRCZ 300 mg/day, the trough concentration decreased to 1.1 µg/mL on day 11 after dose reduction, while CRP declined to 4.78 mg/dL after antibiotic therapy. VRCZ was continued at the same dose. However, four weeks after CRP levels had stabilized, the VRCZ concentration decreased to 0.6 µg/mL. After re-escalation of VRCZ to 400 mg/day, the trough concentration returned to the effective therapeutic range of 1.1 µg/mL.

**Conclusions:**

After infection is controlled, VRCZ concentrations may decrease to levels below the therapeutic range. Following VRCZ dose reduction during a high inflammatory state, reassessment of trough concentrations after improvement of inflammation may be useful to prevent excessive decreases in VRCZ concentrations.

## Background

Invasive pulmonary aspergillosis is an opportunistic infection that occurs in immunosuppressed patients, including those undergoing hematopoietic stem cell transplantation and organ transplantation. Voriconazole (VRCZ) is the first-line antifungal agent for the treatment of invasive and chronic pulmonary aspergillosis [1–3]. VRCZ is metabolized in the liver by the cytochrome P450 (CYP) isoenzymes, primarily CYP2C19, CYP2C9, and CYP3A4 [4]. VRCZ has a narrow therapeutic range; therefore, therapeutic drug monitoring–guided dose adjustment is recommended to ensure efficacy and safety [5]. The effective trough blood concentration of VRCZ is generally considered to be ≥ 1.0–2.0 µg/mL, whereas concentrations exceeding 4–5 µg/mL are associated with an increased risk of liver dysfunction and neurological toxicity [5, 6]. Previous studies have shown that factors contributing to interpatient variability in VRCZ concentrations include age, body weight, concomitant medications (e.g., glucocorticoids and proton pump inhibitors), inflammatory status, liver function, and CYP2C19 genetic polymorphisms [7, 8].

Active infection induces systemic release of inflammatory cytokines, including interleukin-6 (IL-6), interleukin-1β (IL-1β), and tumor necrosis factor-α [9]. These cytokines suppress the activity of hepatic metabolic enzymes, CYP3A4 and CYP2C19, through regulation of transcription factors in the liver [9, 10]. C-reactive protein (CRP) is widely used as a marker of the severity of inflammation and is regarded as a surrogate indicator of the acute-phase response, because its expression is regulated by IL-6–dependent transcription [11]. VRCZ concentrations exhibit both interpatient and intrapatient variability, which correlate with CRP levels [12–15]; van Wanrooy and colleagues reported that the trough concentration of VRCZ increases by 0.015 mg/L for each 1-mg/L increase in CRP [12]. However, inflammatory status varies over time, and improvement in inflammation may alter the concentration of VRCZ. To date, limited attention has focused on the management of VRCZ therapy following the resolution of inflammation.

Here, we report the time-course changes in VRCZ blood concentrations after inflammation in a patient with invasive pulmonary aspergillosis.

## Case presentation

**Fig. 1:**
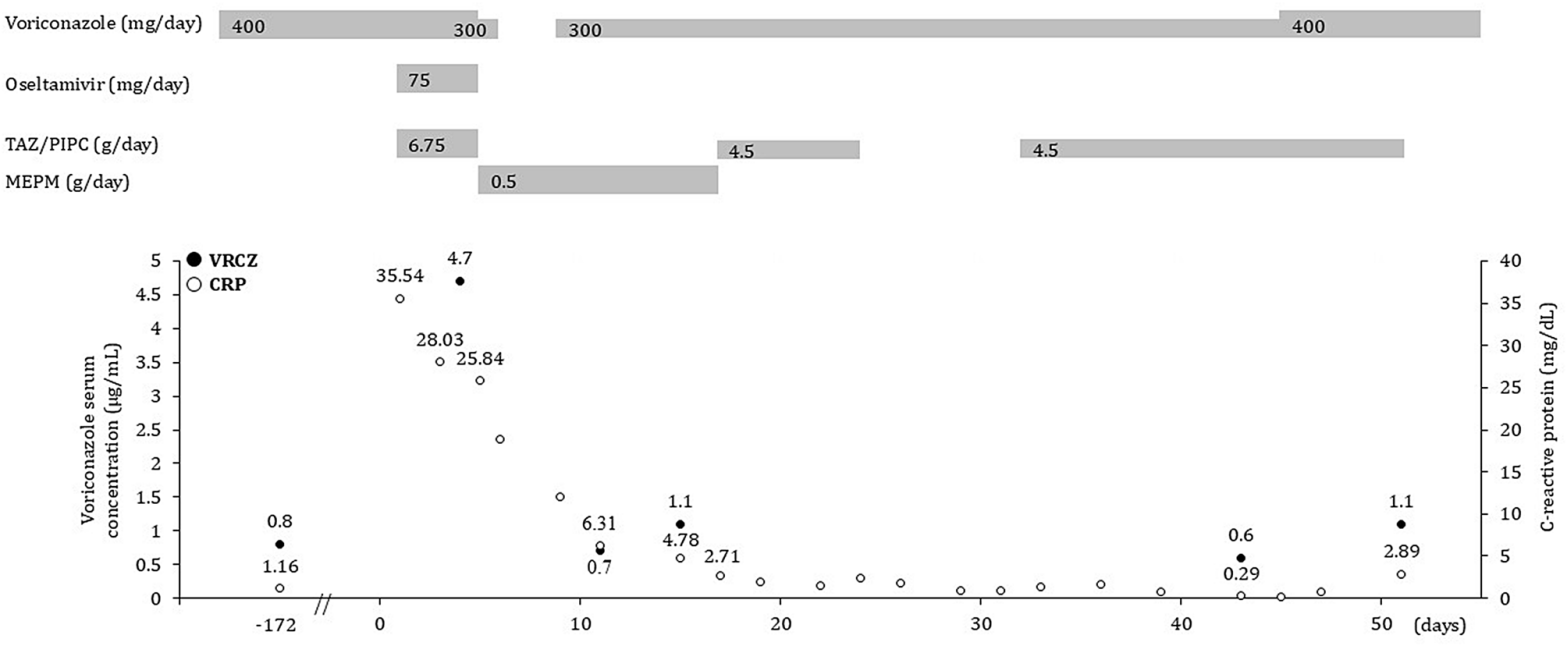
Clinical course of the patient. The doses of voriconazole (VRCZ), oseltamivir, tazobactam/piperacillin (TAZ/PIPC), and meropenem (MEPM), as well as blood VRCZ concentrations (black circles) and C-reactive protein (CRP; open circles), are shown

A 55-kg man in his 60s had developed pulmonary aspergillosis two years prior and had been receiving maintenance therapy with oral VRCZ tablets at 400 mg/day. The dose-normalized concentration (C/D ratio per body weight) was calculated to account for changes in dose and body weight during the clinical course. The most recently measured VRCZ trough concentration was 0.8 µg/mL (C/D ratio: 0.11 [µg/mL]/[mg/kg]) and was considered acceptable in the clinical context (Fig. 1), as both the serum *Aspergillus* galactomannan antigen level and serum β-D-glucan value were stable. At that point, the CRP level was 1.16 mg/dL. His medical history included myelodysplastic syndrome, for which he had undergone bone marrow transplantation five years prior and was currently in remission. Comorbid conditions included membranous nephropathy with nephrotic syndrome, nontuberculous mycobacterial disease, angina pectoris, and hypertension. Concomitant medications consisted of warfarin 2.5 mg/day, furosemide 80 mg/day, tolvaptan 7.5 mg/day, clarithromycin (CAM) 400 mg/day, azilsartan 20 mg/day, and amlodipine 10 mg/day. He was not receiving corticosteroids. Renal dysfunction was present; the serum creatinine level was 2.01 mg/dL, with an estimated glomerular filtration rate based on serum creatinine (eGFRcre) of 27.3 mL/min/1.73 m².

**Fig. 2:**
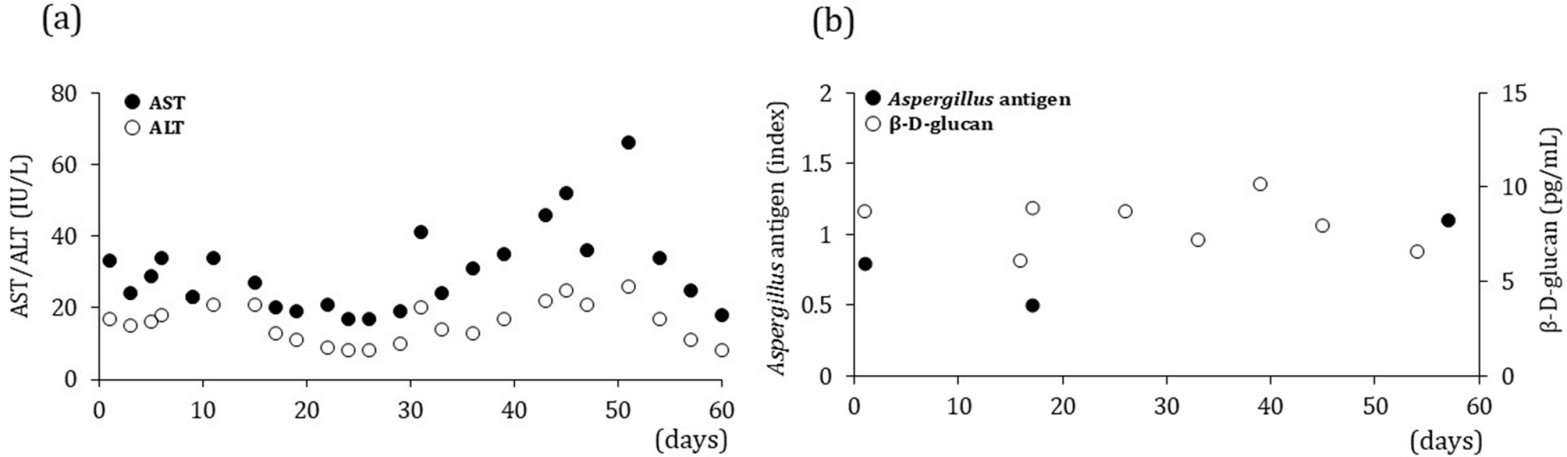
Changes in serum transaminases and fungal biomarkers over time. (**a**) Time course of aspartate aminotransferase (AST; black circles) and alanine aminotransferase (ALT; open circles), (**b**) Time course of *Aspergillus* antigen (black circles) and β-D-glucan (open circles)

On day 1, the patient developed dyspnea and was admitted with suspected bacterial pneumonia and influenza virus infection, which was confirmed by a positive rapid diagnostic test for influenza. Laboratory findings indicated infection, with CRP elevated to 35.54 mg/dL and procalcitonin to 1.20 ng/mL. Laboratory tests showed normal aspartate aminotransferase (AST) and alanine aminotransferase (ALT) levels (33 and 17 U/L, respectively). Figure 2(a) shows the time course of AST and ALT. The creatinine level was 2.86 mg/dL, and the albumin level was 1.5 g/dL. Urinalysis showed proteinuria (3+), and the urinary protein-to-creatinine ratio was 15.1 g/gCr. Serum total cholesterol was 115 mg/dL, LDL cholesterol was 39 mg/dL, and triglycerides were 133 mg/dL. The patient was treated with oseltamivir at 75 mg/day for influenza virus infection and tazobactam/piperacillin (TAZ/PIPC) at 6.75 g/day for suspected bacterial pneumonia. On day 3, CRP level remained elevated at 28.03 mg/dL. The next day, the VRCZ trough concentration was elevated to 4.7 µg/mL (C/D ratio: 0.68 [µg/mL]/[mg/kg]), resulting in a dose reduction to 300 mg/day (Fig. 1). The patient experienced no central nervous system or hepatic disorders related to VRCZ, and no arrhythmias or QT prolongation–related symptoms were observed during concomitant CAM. On Day 5, CRP level remained high at 25.84 mg/dL, and the infection was considered severe; therefore, antimicrobial therapy was changed from TAZ/PIPC to meropenem (MEPM) 0.5 g/day. On Day 6, albumin level was markedly reduced to 0.9 g/dL, and 25% albumin was administered at 12.5 g/day, followed by an increased dose of 25 g/day for two days. The patient experienced a throat obstruction sensation on the same day, temporarily skipped VRCZ, and resumed treatment on Day 9. On Day 11 (seven days after dose reduction), the VRCZ trough concentration had decreased to 0.7 µg/mL (C/D ratio: 0.14 [µg/mL]/[mg/kg]). On Day 15 (eleven days after dose reduction), the VRCZ concentration reached the therapeutic range at 1.1 µg/mL (C/D ratio: 0.22 [µg/mL]/[mg/kg]), and VRCZ therapy was continued at 300 mg/day; the CRP level at that time was 4.78 mg/dL (Fig. 1). MEPM demonstrated marked clinical efficacy, and after CRP decreased to 2.71 mg/dL on Day 17, and the inflammatory state was considered to have stabilized, antimicrobial therapy was switched to TAZ/PIPC 4.5 g/day and discontinued on Day 25.

On Day 30, chest computed tomography revealed an increase in pleural effusion, and thoracic drainage confirmed the presence of purulent pleural fluid. Treatment with TAZ/PIPC at 4.5 g/day led to improvement of the empyema, and by Day 43, the CRP level had decreased to 0.29 mg/dL. Approximately four weeks had passed since CRP stabilization; the serum creatinine level was 2.15 mg/dL, and the albumin level showed an improving trend at 2.2 g/dL. On that day (Day 43), the VRCZ trough concentration again fell below the therapeutic range, measuring 0.6 µg/mL (C/D ratio: 0.10 [µg/mL]/[mg/kg]), which led to re-escalation of the VRCZ dose to 400 mg/day (Fig. 1). CAM was continued at the same dose throughout this period. On Day 51 (seven days after dose escalation), the VRCZ trough concentration had reached the therapeutic range at 1.1 µg/mL (C/D ratio: 0.14 [µg/mL]/[mg/kg]). On the same day, TAZ/PIPC was discontinued due to suspected TAZ/PIPC-induced liver enzyme elevation, and AST and ALT levels decreased gradually. Throughout the hospitalization period, the serum *Aspergillus* antigen index remained between 0.5 and 1.1, whereas serum β-D-glucan concentrations ranged from 6.1 to 10.2 pg/mL (Fig. 2(b)).

## Discussion

In the present case, VRCZ blood concentrations showed delayed changes relative to CRP levels during infection-associated inflammation. Notably, a reduction in VRCZ concentrations was observed four weeks after CRP levels had stabilized. Improvement in the inflammatory state may lead to decreased VRCZ concentrations, potentially resulting in levels below the target therapeutic range. This report highlights the importance of recognizing that subsequent dose escalation of VRCZ may be necessary following resolution of inflammation.

The major circulating metabolite formed through N-oxidation of VRCZ is generated primarily by CYP2C19 [16]. Previous studies have shown that exposure of human hepatocytes to inflammatory cytokines, including IL-6 and transforming growth factor-β, reduces the mRNA expression of CYP2C19 and CYP3A4 [10]. Moreover, CRP concentrations have been reported to correlate with the VRCZ metabolic ratio (VRCZ N-oxide concentration/VRCZ concentration) [17]. Accordingly, the increased CRP levels observed were likely associated with reduced enzymatic activity of CYP2C19 and CYP3A4, leading to increased VRCZ exposure.

On the other hand, the reduction in CRP level due to improved inflammatory status recovers the CYP activity. Previous reports showed that in patients with SARS-CoV-2 infection, the mean CRP level during infection was 91.7 ± 44.6 mg/L, whereas the baseline CRP level three months later was 2.4 ± 1.9 mg/L, and the CYP2C19 metabolic ratio increased by 74.7% [18]. In patients who underwent elective hip surgery, CRP levels decreased and CYP2C19 activity increased between postoperative days 2 and 6, although recovery to normal levels was not observed [19]. Furthermore, while the CRP level peaked on postoperative day 2, CYP2C19 activity decreased from baseline postoperatively, reaching its lowest point on postoperative day 3 [19]. This suggests that a delay may also occur between the decrease in inflammatory cytokine levels and the recovery of CYP activity. In this case, VRCZ 300 mg/day was restarted on day 9, and the VRCZ concentration was 0.7 µg/mL on day 11, primarily reflecting the temporary interruption. The VRCZ concentration subsequently increased to 1.1 µg/mL. Given that VRCZ generally reaches steady state within approximately 5 days [4], the VRCZ concentration was considered to have stabilized. However, approximately 4 weeks later, it decreased to 0.6 µg/mL, while the CRP level declined to 0.29 mg/dL. Since VRCZ and CAM, a CYP3A4 inhibitor, were continued at the same dose during this period, this helped us interpret the clinical course. It is likely that CYP activity recovered to normal levels, leading to increased VRCZ metabolism. These findings, showing a decrease in VRCZ concentrations following a reduction in CRP levels, are consistent with and supported by previous reports [12, 20, 21]. Xiling Jiang et al. reported, based on physiologically based pharmacokinetic (PBPK) model–based simulations, that continuous exposure to IL-6 at 5–100 pg/mL required 7–19 days for hepatic enzyme levels, such as CYP2C19, to reach 90% of a new steady state [22]. After inflammation improved and IL-6 stimulation ceased, the recovery of enzyme levels to the new steady state depended on the turnover rate, characterized by the degradation rate constant (k_deg, H–i_) [22]. In our case, a decrease in VRCZ concentration was observed approximately 4 weeks after CRP levels declined. This time course suggests that CYP2C19 activity had largely returned toward baseline during this period. Therefore, reducing the VRCZ dose during a state of high inflammation may suggest the need for reassessment of VRCZ trough concentrations after CRP levels decrease.

In our case, the patient had hypoalbuminemia associated with nephrotic syndrome, and the infection was considered to have caused an increase in proteinuria, leading to progression of hypoalbuminemia and deterioration of renal function. Hypoalbuminemia is generally associated with an increase in the unbound fraction, leading to increased clearance and decreased total drug concentration [23, 24]. VRCZ is a drug with nonlinear pharmacokinetics [25]. Despite its potential influence on VRCZ protein binding and unbound concentrations, hypoproteinemia has been reported not to affect total VRCZ concentrations [26]. The observed increase in total VRCZ concentrations was therefore likely largely attributable to reduced VRCZ clearance resulting from infection-related suppression of CYP activity rather than from changes in the free fraction. Despite the presence of nephrosclerosis with chronic renal dysfunction, renal function was unlikely to affect VRCZ pharmacokinetics, as only approximately 2% of unchanged VRCZ is excreted in the urine [4].

This case report has limitations. First, we used CRP instead of IL-6. CRP is synthesized in response to stimulation by proinflammatory cytokines, including IL-6 [11]. Moderate correlations have been reported between VRCZ concentrations and both IL-6 and CRP concentrations (IL-6: *r* = 0.46, *P* < 0.0001; CRP: *r* = 0.53, *P* < 0.0001) [27]. In contrast to IL-6, an inflammatory cytokine that is not routinely measured, CRP is a readily available inflammatory marker and is widely used in routine clinical practice. Procalcitonin, an inflammatory marker, was measured only once during treatment; therefore, its utility as a marker of VRCZ pharmacokinetics could not be evaluated. In addition, CYP2C19 genetic polymorphisms were not analyzed in this case. CYP2C19 polymorphisms influence VRCZ concentrations [28, 29]. However, it remains unclear whether they affect the time course of CYP activity recovery. Furthermore, α1-acid glycoprotein (α1-AGP) was not measured in the present case. In patients with markedly reduced albumin concentrations, α1-AGP may have a greater impact on the protein binding of VRCZ [26]. Previous studies have reported a correlation between α1-AGP and total VRCZ concentrations, along with CRP [30]. Therefore, infection-related increases in α1-AGP may have exerted a negative effect on VRCZ concentrations in this case.

## Conclusion

This case report suggests that improvement in the inflammatory state may be associated with increased VRCZ metabolism, resulting in VRCZ concentrations below the therapeutic range. Following VRCZ dose reduction during a high inflammatory state, reassessment of trough concentrations after inflammatory improvement, with subsequent dose adjustment, may be useful to prevent excessive decreases in VRCZ concentrations.

## Data Availability

All data generated or analyzed during this study are included in this published article.

## Abbreviations

α1-AGP: α1-acid glycoprotein
ALT: Alanine aminotransferase
AST: Aspartate aminotransferase
CAM: Clarithromycin
CRP: C-reactive protein
CYP: Cytochrome P450
IL-6: Interleukin-6
MEPM: Meropenem
TAZ/PIPC: Tazobactam/piperacillin
VRCZ: Voriconazole
